# Acknowledgment of *Ad Hoc* Reviewers

**DOI:** 10.1128/mSphere.00873-19

**Published:** 2019-12-18

**Authors:** Michael J. Imperiale

**Affiliations:** a University of Michigan Medical School, Ann Arbor, Michigan, USA

## EDITORIAL

2019 was another year of growth for *mSphere*: our numbers of submitted and published manuscripts increased substantially over last year. The high quality of the work that we have published is reflected in how well these articles are being cited in the literature. While this increase has meant more throughput for our senior editors, editors, and staff, we have maintained our commitment to quality and to rapid decision-making. As always, we could not adhere to these standards without the substantial effort provided by those of you who served as reviewers. Your thoughtful and fair guidance to our authors improves the quality of the science and benefits our community tremendously. We thank all of you who have contributed your time to *mSphere* in 2019.

Kjersti Aagaard
Sophie Saphia Abby
Elsayed M. Abdelwhab
Steven Abel
James Adams
Mark D. Adams
Getahun E. Agga
Warish Ahmed
Tatsuya Akiyama
Denise M. Akob
Oleg Alexeyev
Howbeer Muhamad Ali
Michael S. Allen
Emma Allen-Vercoe
Francis Alonzo III
Daniel Amador-Noguez
Stefano Amalfitano
Jorge Amich
Karthik Anantharaman
Annaliesa S. Anderson
David R. Andes
Raul Andino
Elliot J. Androphy
Medini K. Annavajhala
Haike Antelmann
Sergio Arce
Matthew J. Arduino
Zach Armstrong
Paul Ashwood
Jennifer M. Auchtung
Clara M. Ausiello
Koichiro Awai
Frank O. Aylward
M. Andrea Azcarate-Peril
Catherine Bachewich
Alexandre Bagnoud
Alexa Bakker
Megan Tierney Baldridge
Elizabeth Ballou
Jeffrey A. Banas
James D. Bangs
Saumya Bansal
Mariette Barbier
Francois-Xavier Barre
Eileen M. Barry
Ryan P. Bartelme
Jason C. Bartz
Susan Baserga
Christine Marie Bassis
Miles Hume Beaman
Andrea Beaton
Peter Trip Beernink
Michael Behnke
Peter Belenky
Vivian Bellofatto
William H. Benjamin
Sarah Ben Maamar
Kara A. Bernstein
Gregory J. Berry
Stefan Bertilsson
Bryan J. Berube
Purnima Bhanot
Fadil A. Bidmos
Piotr Bielecki
Jacob P. Bitoun
Ryan Andrew Blaustein
Jesus Blazquez
Pierre Bogaerts
Matthew Bogyo
Raquel Regina Bonelli
German Bonilla Rosso
Remy A. Bonnin
Adrianus C. Boon
Travis Bourret
Teun Bousema
Jon P. Boyle
Kenneth A. Bradley
Melanie Bressan
Rebeccah Brewer
William J. Britt
Robert A. Britton
Daniel R. Brown
Jeremy S. Brown
Kevin M. Brown
Roberto Emanuel Bruna
Shilpa Buch
Wolfgang Buckel
Laurel S. Burall
Saul Burdman
Cara C. Burns
Michael Burns
Niels Burzan
Karen Bush
Sarah N. Buss
Henning Büttner
David J. Bzik
Cassandra M. Campion
Alessandra Carattoli
Franck Gael Carbonero
Paul Carini
Ronan K. Carroll
Vern B. Carruthers
Dee A. Carter
Cecilia G. Carvalhaes
Ana Paula D’Alincourt Carvalho-Assef
Leslie S. Casey
Mariana Castanheira
Jeff L. Caswell
Rodrigo Cayô
Louise Cerdeira
Yunrong Chai
Josephine R. Chandler
Theresa Chang
Pascale G. Charest
Delphi Chatterjee
Sujata S. Chaudhari
Rama Chaudhry
Jenn-Wei Chen
Jianfei Chen
Jun Chen
Leonid V. Chernomordik
Konstantin Chumakov
Christine Clayton
Joachim Clos
William L. Close
Ian Cockburn
Tom Coenye
Sarah A. Collier
Lydia M. Contreras
Tyrrell Conway
Laura C. C. Cook
Kerri Coon
Peggy A. Cotter
Peter V. Coyle
Dean C. Crick
Paul J. Cullen
Gordon Fritz Custer
Sezin Dagdeviren
James B. Dale
Julian Damashek
Remus Dame
Joshua B. Daniels
Paban Kumar Dash
Dana A. Davis
Gregg S. Davis
George S. Deepe
Elizabeth N. De Gaspari
Kirk W. Deitsch
Frank R. DeLeo
Maurizio Del Poeta
Eric L. Delwart
Tom Johannes Bernardus de Man
Hasan Demirci
Jigar V. Desai
Lalitagauri Deshpande
Santosh Dhakal
Don J. Diamond
Robert P. Dickson
Marta Diez-Valcarce
Joseph P. Dillard
Pedro Dimitriu
Julianne T. Djordjevic
Andrzej A. Dlugosz
Maria G. Dominguez
Maureen J. Donlin
Zhicheng Dou
Benoît Doublet
Cynthia S. Dowd
Kirk Druey
Yehui Duan
John Dunn
Rachel J. Dutton
Jeffrey D. Dvorin
Hari Dwivedi
Mark Ebell
Mariola J. Edelmann
Mira Edgerton
Adrian Egli
Jeremy R. Ellermeier
David M. Engman
Armin Ensser
Lauren Epstein
Hasier Eraña
Marina Eremeeva
Juan-Carlos Espinosa
Tom J. Evans
Seamus Fanning
Paula J. Fernandes
Rachel C. Fernandez
Susana Ferreira
Michael Fiechtner
Mark C. Field
Scott G. Filler
Jamie Findlow
Kevin Thomas Finneran
David A. Fitzpatrick
Jason P. Folster
Thierry Fontaine
Jarrod R. Fortwendel
James G. Fox
Michael France
Nelson Frazão
Stephen J. Free
Nancy E. Freitag
Olivier Fritsch
Aline Frossard
Ping Fu
Ernesto J. Fuentes
Jennifer Fulcher
Genevieve G. G. Fouda
Attila Gacser
Sreenivas Gannavaram
Danielle A. Garsin
Beth A. Garvy
Justus Garweg
Anne A. Gershon
Karine A. Gibbs
Jason Gigley
Sabine Gilch
Carol A. Gilchrist
Steven R. Gill
Joseph James Gillespie
Patrick M. Gillevet
Delphine Girlich
Elisabetta Giuffra
Douglas Paul Gladue
Eva Gluenz
Henry P. Godfrey
Joachim Goedhart
Boris Goerke
Gustavo H. Goldman
Mark Gomelsky
Richard H. Gomer
Maria Gomes-Solecki
Andres Gomez
Jorge Enrique Gómez-Marín
Ryota Gomi
Tobias Goris
Stephan Göttig
Guillaume Goyette-Desjardins
Patricia Grace
Peter L. Graumann
Scott D. Gray-Owen
Kim Y. Green
E. Peter Greenberg
Isabella Gremião
Alexander L. Greninger
Richard L. Guerrant
Antonieta Guerrero-Plata
Mohan Gupta
John E. Gustafson
Richard Haaland
Timmothy Hackmann
Ted Hackstadt
Julia Cornelia Johanna Petronella Hagenaars
Constantine G. Haidaris
Nancy L. Haigwood
Hillel Haim
Mohamed-Ali Hakimi
Luanne Hall-Stoodley
Scott B. Halstead
Neal D. Hammer
Matthew Hansen
Nancy D. Hanson
Christopher Harmer
Joe Harrison
Eric T. Harvill
Cynthia Y. He
Tonje Marita Bjerkan Heggeset
Henry S. Heine
David E. Heinrichs
Jon Heinrichs
Betsy Herold
Anat A. Herskovits
Dagmar Heuer
Kevin Loren Hockett
Richard L. Hodinka
Anders Hofer
Matthew T. G. Holden
Michael J. Holmes
Peiying Hong
Alexander R. Horswill
Joppe Willem Hovius
Fupin Hu
Shi Huang
Ruth Huizinga
Dana E. Hunt
Robert P. Hunter
A. Hurley
Holly K. Huse
Naoki Inoue
Anna C. Jacobs
Maritza Jaramillo
Armando Jardim
Keith William Jarosinski
Vicki Jeffers
Seok Hoon Jeong
Xiaofei Jiang
Veronica Jimenez
Christian Jobin
Andrew W. Johnston
Bradley D. Jones
Clinton J. Jones
Howard S. Judelson
Sheryl Justice
Mehdi Kabbage
Mary E. Kable
David Kadosh
Lindsay Kalan
Hyun Ah Kang
Maria Karlsson
Christina Karsten
Thomas E. Kehl-Fie
Brendan Kelly
Arifa S. Khan
Sarah E. Kidd
Jonathan Klassen
Kimberly A. Kline
Laura J. Knoll
Michael H. Kogut
Tse Hsien Koh
James B. Konopka
Nicole Marie Koropatkin
Konstantin G. Kousoulas
Renato Kovacs
Thomas R. Kozel
Florian Krammer
Barry N. Kreiswirth
Jens Kreth
Lee Kroos
Kengo Kubota
Ulrich Kück
Louise Kuhn
Ritwij Kulkarni
Anuj Kumar
Ashok Kumar
Limin Kung
Hiroyuki Kusada
Joseph L. Kuti
Matthias Labrenz
Kaice Lafavers
Laimonis A. Laimins
Noelia Lander
Santo Landolfo
Gordon Langsley
Paweł Łaniewski
Peter Larson
Gee W. Lau
Adam S. Lauring
Anton Lavrinienko
Steven Lawson
Simon Lax
Carlo Lazado
Vladimir Lazarevic
Helen M. Lazear
Sarah Lebeer
Yoann Le Breton
Chia Y. Lee
Kyungwon Lee
Sangmi Lee
Soo Chan Lee
John Lees
McKenzie K. Lehman
Gabriela G. S. Leite
Jose A. Lemos
Cammie F. Lesser
Gabriel Leventhal
David E. Levin
Deborah Lewinsohn
Ruichao Li
Ziyin Li
Yuying Liang
Stephen J. Libby
Dominique Limoli
Xiaorong Lin
Nilton Lincopan
Gunnar Lindahl
Inge Lindseth
Dirk Linke
Alexandra M. Linz
Marc Lipsitch
Trevor Lithgow
Elizabeth Littauer
Bao-Tao Liu
Cindy M. Liu
Jian-Hua Liu
Kenneth J. Locey
Melissa Bruckner Lodoen
Olga Lomovskaya
John M. Lopes
Job E. Lopez
Carolina López
Ashley Jane Losier
Catherine Lozupone
Lenette Lu
Jost Ludwig
Micah A. Luftig
Julius Lukeš
Joseph Daniel Lutgring
Douglas S. Lyles
Bing Ma
Jiyan Ma
Li-Jun Ma
Zachary B. Mackey
Jean-Yves Madec
Elizabeth A. Maga
Michael J. Mahan
Iris Maldener
Reet Mändar
Francois Frederick Maree
Kevin Maringer
Sara Marti
Willames M. B. S. Martins
Karl R. Matthews
Andreas Mayer
Joseph McBride
John K. McCormick
Larry S. McDaniel
Daniel McDonald
Megan McEvoy
Paul McGovern
James B. McKinlay
Joel McManus
Spyridon Megremis
Andrew Mehle
Jacquelyn S. Meisel
Roberto G. Melano
David A. Melnick
Paola E. Mera
Craig Meyers
Shulamit Michaeli
Gurvan Michel
Dejan Micic
Alita A. Miller
Matthew S. Miller
Kathryn Milligan-Myhre
Kingston H. Mills
Kyunghun Min
Maria F. Mojica
Claudia R. Molins
Vicente Monedero
Robert W. Moon
Gary Moran
Hiroshi Mori
Yuki Morono
Naomi Morrissette
Joachim Morschhäuser
Gregory William Moseley
Quézia Moura
Jarrod J. Mousa
W. Scott Moye-Rowley
Monica Mugnier
David Muller
Matthew A. Mulvey
Kostas Mumcuoglu
Karl Münger
Jose M. Munita
Sharmishtha Musalgaonkar
Peter Myler
Ian A. Myles
Indira U. Mysorekar
Lisbeth M. Naess
Moon H. Nahm
Farooq Nasar
David M. Needham
John D. Neill
Eric Jorge Nelson
Jeniel E. Nett
Stephanie L. Neville
Irene L. G. Newton
M. Hong Nguyen
Wright W. Nichols
Pablo Ivan Nikel
S. Nikkari
Daniel R. Noguera
Lisa Nonaka
Romolo Nonno
Steven J. Norris
Eva Novakova
Tomoyoshi Nozaki
Austin S. Nuxoll
Tyler K. Nygaard
Anna Oevermann
Yusuke Okazaki
Mónica Oleastro
Daniel Groban Olson
Michael Olson
Eng Eong Ooi
Andres Opazo-Capurro
Manuel Osorio
Gustavo Palacios
John C. Panepinto
Kevin Panke-Buisse
Costas C. Papagiannitsis
John R. Papp
D. Joshua Parris
Colin R. Parrish
Sally R. Partridge
Jean B. Patel
Samir N. Patel
Wilhelm Paulander
Martin S. Pavelka
Mark E. Peeples
Catia Taniela Perciani
Ines C. Pereira
Daniel R. Perez
Federico Perez
Guillermo I. Perez-Perez
Stanley Perlman
Vincent Perreten
Renée M. Petri
Stephanie Pfaender
William Picking
Maurice Pitesky
Johann Pitout
Laurent Poirel
Michael Poulsen
Rafael Prados-Rosales
Rachel Presti
Michael William Pride
Harry E. Prince
Caitlin Proctor
Steven J. Projan
Janet Quinn
Nancy Raab-Traub
Matthew Ralls
Mario Ramirez
Reeta Prusty Rao
Chad A. Rappleye
David Rasko
Kasie Raymann
Timothy D. Read
Michael B. Reed
Matthew Reeves
Todd B. Reynolds
Kelly C. Rice
Bassam Rimawi
Christine Hanssen Rinaldo
Florence Robert-Gangneux
Ignasi Roca
César Rodríguez
F. Roesl
Tom Rogers
Robin Rebecca Rohwer
Ronda J. Rolfes
Thierry Rolling
Sandra Romero-Steiner
Francoise Helene Routier
Ludmila V. Roze
Luis A. Rubio
Natividad Ruiz
David A. Sack
Helio S. Sader
Jeroen P. J. Saeij
Manish Sagar
Nita H. Salzman
John C. Samuelson
Ezequiel Santillan
Viviane Santos De Sousa
Per Erik Joakim Saris
Christopher Sassetti
Xavier Sastreg
Karla J. F. Satchell
Kei Sato
Karin Sauer
Joy Scaria
Patrick M. Schlievert
Patrick D. Schloss
Marian L. Schmidt
Günther Schönrich
Tony Schountz
William R. Schwan
Anna Maria Seekatz
H. Steven Seifert
Keun Seok Seo
William M. Shafer
Rebecca S. Shapiro
Zhangqi Shen
Ameet Shetty
Baochen Shi
Rima Shrestha
Paul Sigala
Ellen K. Silbergeld
Lynn L. Silver
Patricia J. Simner
Augusto Simoes-Barbosa
Anthony P. Sinai
Thaís Cristine Marques Sincero
Amit Singh
Kamesh Sirigireddy
Mark S. Smeltzer
Celia Maria Almeida Soares
Marcelo A. Soares
Heidi Marie Soeters
Hokyoung Son
Grace A. Spatafora
Jessica R. Spengler
Igor Splichal
Michelle Spoto
Prakash Srinivasan
Peter Staeheli
Kenneth A. Stapleford
Ann E. Stapleton
Eliana Stehling
Brian Stevenson
David B. Stewart
O. Colin Stine
Milos Stojanov
Gregory G. Stone
Paul Stoodley
Sailendharan Sudakaran
Yongjun Sui
Nicole L. Sullivan
Michael G. Surette
Fayyaz Sutterwala
Yasuhiro Suzuki
Birte Svensson
Balazs Szoor
Diana Hazard Taft
Kaitlin A. Tagg
Hiroki Takahashi
Hengli Tang
Fernando Tavares
Michael E. Taveirne
Miguel Cacho Teixeira
Lesly A. Temesvari
Ken Teter
Kevin R. Theis
Teresa Thiel
Vinai Chittezham Thomas
Krystal Thomas-White
Andrew Tomaras
Hung Ton-That
Begum D. Topcuoglu
Karen Trchounian
Kathleen Treseder
Stephen Tristram
Can Murat Ünal
Gottfried Unden
Wendy W. J. Unger
Priya Uppuluri
Syun-Ichi Urayama
David W. Ussery
Tamara van Gorkom
Willem van Schaik
Daria Van Tyne
Arvind Varsani
Vittorio Venturi
Akash H. Verma
James Versalovic
Birte Vester
Adrien Vigneron
Christian Vinueza
Coralie Viollet
Emily Vogtmann
Jatin M. Vyas
Joseph Thomas Wade
Seth T. Walk
Daniel Wall
David A. Walsh
Christian W. Wang
David Wang
Guangshun Wang
Huanyu Wang
Ping Wang
Tony Wang
Yang Wang
Honorine D. Ward
Matthew J. Wargo
Emma Wear
Keith E. Weaver
Mary Weber
Louis M. Weiss
Jonathan Weitzman
Rodney A. Welch
Melanie Wellington
Christopher West
Stephen C. Whisson
Stephen S. Whitehead
Shannon Whitmer
Thomas Wiegert
Michael Wilkins
Brian J. Wilkinson
Freyja Williams
John V. Williams
Emma H. Wilson
Richard A. Wilson
Christiane E. Wobus
Alan J. Wolfe
Adam C. N. Wong
Kate A. Worthing
Karen Wozniak
Rachel A. F. Wozniak
Brendan W. Wren
Terry W. Wright
Kristine M. Wylie
Kelly Wyres
Hang Xie
Jin-Rong Xu
Kyosuke Yamamoto
Shinji Yamasaki
S. Steve Yan
Xiang Yang
Hui-Ling Yen
Jeremy A. Yethon
Xiaochen Yin
Yanbin Yin
Xi Yu
Ze-Chun Yuan
Joseph P. Zackular
Allan Zajac
Fillipo Zambelli
Raffaele Zarrilli
Qixiao Zhai
Qijing Zhang
Jin Zhong
Dejian Zhou
Yanjiao Zhou
Baoli Zhu
Wenfei Zhu
Dan Zilberstein
Stephen H. Zinder
Erik R. Zinser
Sadri Znaidi
Susan B. Zolla-Pazner

